# Propensity score analysis of lung cancer risk in a population with high prevalence of non-smoking related lung cancer

**DOI:** 10.1186/s12890-017-0465-8

**Published:** 2017-09-06

**Authors:** Kuei-Feng Lin, Hsiu-Fu Wu, Wei-Chun Huang, Pei-Ling Tang, Ming-Ting Wu, Fu-Zong Wu

**Affiliations:** 1 Department of Radiology, Kaohsiung Veterans General Hospital, Kaohsiung, Taiwan; 2 Faculty of Medicine, School of Medicine, Institute of Clinical Medicine, National Yang Ming University, Taipei, Taiwan; 3 Research Center of Medical Informatics, Kaohsiung Veterans General Hospital, Kaohsiung, Taiwan; 4 School of Medicine, College of Medicine, Kaohsiung Medical University, Kaohsiung City, Taiwan

**Keywords:** Non-smoker lung cancer, Propensity score matching, Lung adenocarcinoma spectrum, Risk factor

## Abstract

**Background:**

Lung cancer has been the leading cause of cancer-related mortality worldwide among both men and women in recent years. There is an increase in the incidence of nonsmoking-related lung cancer in recent years. The purpose of the present study was to investigate multiple potential risk factors for nonsmoking-related lung cancer among Asian Ethnic Groups.

**Methods:**

We used a propensity score-mated cohort analysis for this study. We retrospectively review the medical record of 1975 asymptomatic healthy subjects (40 ~ 80 years old) who voluntarily underwent low-dose chest CT from August 2013 to October 2014. Clinical information and nodule characteristics were recorded.

**Results:**

A propensity score-mated cohort analysis was applied to adjust for potential bias and to create two comparable groups according to family history of lung cancer. For our primary analysis, we matched 392 pairs of subjects with family history of lung cancer and subjects without history. Logistic regression showed that female gender and a family history of lung cancer were the two most important predictor of lung cancer in the endemic area with high prevalence of nonsmoking-related lung cancer (OR = 11.199, 95% CI = 1.444–86.862; OR = 2.831, 95% CI = 1.000136–8.015). In addition, the number of nodules was higher in subjects with family history of lung cancer in comparison with subjects without family history of lung cancer (OR = 1.309, 95% CI = 1.066–1.607).

**Conclusions:**

In conclusion, risk-based prediction model based on the family history of lung cancer and female gender can potentially improve efficiency of lung cancer screening programs in Taiwan.

## Background

Lung cancer has been the leading cause of cancer-related mortality worldwide among both men and women in recent years [1–3]. The landmark National Lung Screening Trial (NLST) evaluated the benefits of low-dose computed tomography (LDCT) for screening of heavy smokers (≥30 pack-years) and found that annual screening by LDCT yielded a relative reduction of lung cancer mortality of 20% among those screened when compared to chest radiography [3]. Smoking is the major risk factor for lung cancer, but an increase in the incidence of nonsmoking-related lung cancer in recent years has been addressed [4–8]. There has been an increase in the prevalence of non-smoking associated lung cancers in Asian countries such as China, Taiwan, Korea, and Japan over the past few years [9, 10]. Previous studies suggested that a potential association among nonsmokers who had lung adenocarcinoma with associated risk factors such as age, gender, body mass index (BMI), history of lung cancer, and personal cancer history [8, 11]. A major concern has remained regarding that selection bias that occurs as a result of self-referral or physician referral in the setting of these studies designs, which is ordinarily considered a threat to both internal and external validity of the studies [8, 11]. Propensity score matching method is increasingly being used currently and a useful statistical technology in observational studies to ensure that propensity score is balanced across treatment and control groups as an alternative to conventional covariate adjustment in logistic regression models [12]. Using propensity score matching analysis, clinical/demographic characteristics of subjects between the groups with family history of lung cancer (+) versus without family history of lung cancer (−) could be balanced out, thus mimicking randomized controlled trial design. The purpose of the present study was to investigate potential risk factors for nonsmoking-related lung cancer among Asian population based on propensity score matching analysis which could reduce selection bias and potential baseline differences between the two groups.

## Methods

### Study population and cohort

**Fig. 1:**
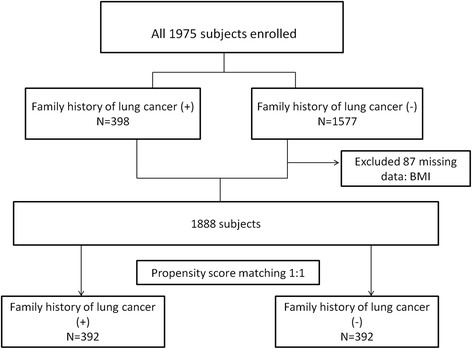
Flowchart with a summary of patient enrollment and propensity score matching

A flow diagram describing the subject recruitment and exclusions is shown in Fig. 1. We retrospectively analyzed 1975 (1083 males and 892 females) asymptomatic healthy subjects (age range 40 to be 80-year-old) who voluntarily underwent self-paid LDCT exam at the health check-up center of Kaohsiung Veterans General Hospital from August 2013 to October 2014. Clinical information included gender, age, BMI, family history of lung cancer, and family history of other cancers in first and second-degree relatives was collected. Moreover, nodular characteristics were recorded according to ACR Lung-RADS classification shown in Table 1 [13, 14]. Categories1 (negative) and 2 (benign appearance) correspond to negative screening results, and categories 3 (probably benign) and 4 (suspicious) correspond to positive screening results. Category 4 is divided into 4A, 4B, and 4X, based on the level of suspicion of malignancy according to the nodule size and characteristics summarized in Table 1. Increases in the probability of malignancy are expressed by assigning either subcategory, 4A (5%–15%), 4B (>15%), or 4X (additional finding such as spiculation or enlarged lymph nodes). The average follow-up time of subjects with suspicious nodules was 1.6 ± 0.5 years after the baseline LDCT.

**Table 1:** Summary of Lung-RADS Classification^a^

Lung-RADS	Baseline screening	Subsequent screening
1	No nodules; nodules with calcification	No nodules; nodules with calcification
2	Solid/part solid: < 6 mm	Solid/part solid: < 6 mm
	GGN: < 20 mm	GGN:< 20 mm or unchanged/slowly growing
		Category 3–4 nodules unchanged at ≥3 mo
3	Solid: ≥ 6 to <8 mm	Solid: New ≥4 to <6 mm
	Part solid: ≥ 6 mm with solid component <6 mm	Part solid: New <6 mm
	GGN: ≥ 20 mm	GGN: New ≥20 mm
4A	Solid: ≥ 8 mm to <15 mm	Solid: Growing <8 mm or new ≥6 and <8 mm
	Part solid: ≥ 8 mm with solid component ≥6 and <8 mm	Part solid: ≥ 6 mm with new or growing solid component <4 mm
4B	Solid: ≥15 mm	Solid: New or growing and ≥8 mm
	Part solid: Solid component ≥8 mm	Part solid: ≥ 6 mm with new or growing solid component ≥4 mm
4X	Category 3 or 4 nodules with additional features; imaging findings that increase suspicion of malignancy	Category 3 or 4 nodules with additional features; imaging findings that increase suspicion of malignancy

*GGN* ground-glass nodule

^a^ Size is the average diameter rounded to the nearest whole number. Growth is a size increase >1.5 mm

Lung-RADS: The ACR Lung Imaging Reporting and Data System

Among 1975 screened subjects, 72.8% (1438/1975) of the screened subjects were never-smokers, 16.5% (326/1975) were current smokers, and 10.7% (211/1975) were former smokers. Only 7.5% (149/1975) of the study subjects would have been eligible for screening based on the NLST enrollment criteria. Among 1975 screened subjects, there were 27 subjects diagnosed with non-smoking related lung cancer (two lung cancer subjects with smoking were excluded). Definition of non-smoking related lung cancer was defined as the lung adenocarcinoma spectrum such as adenocarcinoma in situ, minimally invasive adenocarcinoma, and invasive adenocarcinoma diagnosed by surgical or biopsy proof. Histopathologic diagnosis of atypical adenomatous hyperplasia was excluded from this study.

### Covariate and propensity score matching

All the subjects were divided to two groups: the group with family history of lung cancer (398 subjects) and the group without family history of lung cancer (1577 subjects). However, 87 patients were excluded because of missing data on BMI profiles. We used a 1:1 propensity score-matched pair method combined with covariate adjustment to analyze patients with and without family history of lung cancer shown in Fig. 1. The unbalanced conditions at baseline between the two groups were controlled by using PS matching with covariate adjustment. The 1:1 PS matching yielded matched pairs of 392 subjects with family history of lung cancer and 392 patients without family history of lung cancer, resulting in no differences in age, gender, BMI, and the proportion of other cancers of family history.

### LDCT imaging acquisition and interpretation

All scans were performed with a 16-slice multi-detector CT (Somatom Sensation 16, Siemens Healthcare, Erlangen, Germany) and a 64-slice multi-detector CT (Aquilion 64; Toshiba Medical Systems) from the lung apex to the base without contrast enhancement. The LDCT examination protocols met the CMS (Centers for Medicare & Medicaid Services) requirement of the volume CT dose index (CTDIvol) ≤ 3.0 milligray (mGy) for standard-size patients based on recommendations of the ACR and Society of Thoracic Imaging for different vendors setting [15]. Scans were obtained with the subjects in supine position at end inspiration. The data were reconstructed with filtered back projection, a slice thickness of 2 mm, and an increment of 2 mm, using a smooth convolution kernel (Siemens B30f, Toshiba FC02). All studies were evaluated on lung and mediastinal windows on a picture-archiving and communication system and reported by two experienced thoracic radiologists with 8 and 12 years of experience, respectively.

### Statistical analysis

Statistical analysis was performed using SPSS® v17.0 for Windows (SPSS, Inc., Chicago, IL) and the SAS® software package (SAS Institute, Inc., Cary, NC). To minimize the effect of potential confounders on selection bias, propensity scores were generated by using the multiple logistic regressions to estimate the probability that subjects have family history of lung cancer or not. The covariates entered into the propensity score were age, gender, and BMI. Propensity score matching (1:1 match) was performed to adjust for differences in baseline clinical characteristics, yielding a total of 784 subjects: 392 subjects with family history of lung cancer and 392 subjects without family history of lung cancer (SAS Institute, Inc., Cary, NC).

Baseline characteristics were performed as mean ± standard deviation (SD).

Comparisons between the two groups were performed by using the independent T-test for continuous data and chi-square test for categorical data before and after PS matching. The Fisher exact chi-square test was used to analyze when the smallest expected value is less than 5. Multiple logistic regression models were developed, and odds ratios (ORs) were used to evaluate risk factors associated with lung cancer. Data analysis was performed using SPSS® v17.0 for Windows (SPSS, Inc., Chicago, IL).

## Results

We retrospectively review the medical record of 1975 asymptomatic healthy subjects (40 ~ 80 years old) who voluntarily underwent low-dose chest CT (1083 males, 892 females) from August 2013 to October 2014. We identified 398 patients with family history of lung cancer while the other 1577 patients without family history of lung cancer shown in Fig. 1. The baseline characteristics in the pre-match and post-match cohorts are presented in Table 2.

**Table 2:** Patient characteristics before and after propensity score matching

	Before PSM (*N* = 1975)					After PSM (*N* = 784)				
Characterics	All		Family history (+)	Family history (−)	*P*	All		Family history (+)	Family history (−)	*P*
Age, years	56.56 ± 9.01		56.1 ± 9.39	58.39 ± 7.05	<0.0001^a^	58.61 ± 7.15		58.57 ± 6.855	58.66 ± 7.556	0.865^a^
Sex (%)					<0.0001^b^					0.94^b^
Male	1083	54.90%	136 (34.1%)	947 (60.05%)		517	65.90%	258 (65.8%)	259 (66.1%)	
Female	892	45.10%	262 (65.9%)	630 (39.95%)		267	34.10%	134 (34.2%)	133 (33.9%)	
BMI	24.32 ± 3.49		23.76 ± 3.36	24.46 ± 3.50	<0.0001^a^			23.76 ± 3.36	23.88 ± 3.56	0.644^a^
Nodule number	0.63 ± 1.16		1.09 ± 1.53	0.51 ± 1.027	<0.0001^a^	0.84 ± 1.392		1.1 ± 1.53	0.59 ± 1.17	<0.0001^a^
History of other cancers					0.023^b^					0.601^b^
Present	621	31.40%	144 (36.1%)	477 (30.2%)		275	35.10%	141 (36%)	131(34.2%)	
Absent	1354	68.50%	254 (63.9%)	1100 (69.8%)		509	64.90%	258 (64%)	251(65.8%)	
Category 4 lesion					<0.0001^b^					0.186^b^
Present	53	2.68%	21 (5.27%)	32 (2.02%)		36	4.59%	21 (5.3%)	15 (3.82%)	
Absent	1922	97.32%	377 (94.73%)	1545 (97.98%)		748	95.41%	371 (94.7%)	377 (96.18%)	
Lung cancer					<0.0001^b^					0.019^c^
Present	27	1.40%	15 (3.76%)	12 (0.76%)		20	2.60%	15 (3.8%)	5 (1.3%)	
Absent	1948	98.60%	383 (96.24%)	1565 (99.24%)		764	97.40%	377 (96.2)	387 (98.7)	

^a^Using independent t-test for continuous variables; ^b^ Using Chi-square test for categorical variables; ^c^ Using Fisher’s exact test for categorical variables

Abbreviations: *PSM* propensity score matching, *BMI* body mass index

### Baseline characteristics before propensity matching

Patients were significantly younger in the family history of lung cancer (+) group compared with the family history of lung cancer (−) group (56.1 ± 9.39 years old versus 58.39 ± 7.05 years old); the BMI in the family history of lung cancer (+) group is lower compared with the family history of lung cancer (−) group (23.76 ± 3.36 kg/m^2^ versus 24.46 ± 3.50 kg/m^2^); there were more nodules in the family history of lung cancer (+) group compared with the family history of lung cancer (−) group (1.09 ± 1.53 versus 0.51 ± 1.027). There were several parameters of baseline characteristics statistically higher in the family history of lung cancer (+) group, including the percentage of female gender (65.9% vs. 39.95%), the percentage of category 4 lesions (5.27% vs. 2.02%), the percentage of family history of other cancers (36.1% vs. 30.2%), and the percentage of lung cancer (3.76% vs. 0.76%).

Among 27 subjects with non-smoking related lung cancer diagnosed, 8 (29.62%) subjects had a diagnosis of synchronous multiple primary lung cancers (MPLCs) according to the diagnostic criteria proposed by Martini and Melamed before propensity score matching [16]. Among 20 (35%) subjects with non-smoking related lung cancer diagnosed, 7 subjects had a diagnosis of synchronous MPLCs according to the diagnostic criteria proposed by Martini and Melamed after propensity score matching [16].

**Fig. 2:**
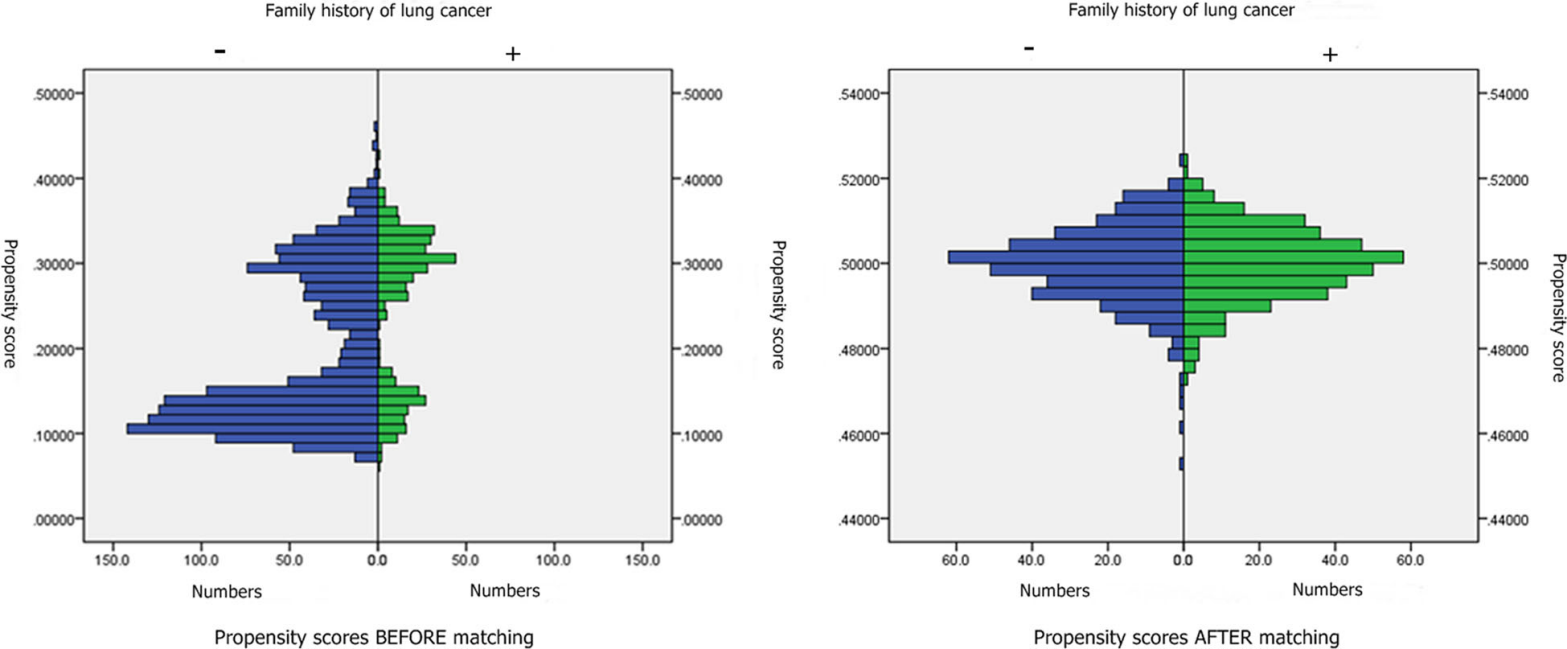
**a**-**b** Histograms of propensity score distribution before and after propensity score matching. Distribution of the propensity scores before and after matching for group of family history of lung cancer (+) and group of family history of lung cancer (−). **a** presents histograms of unbalanced propensity score distribution in both groups before propensity matching. **b** presents histograms of balanced propensity score distribution in both groups after propensity matching

To further investigate this imbalance, we illustrate histogram of the distribution of the propensity score for both groups before and after propensity matching. Figure 2a presents histograms of unbalanced propensity score distribution for both groups before propensity matching. Figure 2b presents histograms of balanced propensity score distribution for both groups after the propensity matching.

### Baseline characteristics after propensity matching

According to the propensity score matching 1:1 shown in Table 2 **,** 392 patients in the family history of lung cancer (+) group were matched with 392 in the family history of lung cancer (−) group. The matching process eliminated some significant differences that existed between the family history of lung cancer (+) group and the family history of lung cancer (−) group such as age, sex, BMI, the percentage of family history of other cancers, and category 4 lesions, while the nodule numbers and the percentage of lung cancer remained significant different.

### Univariate and multivariate logistic regression analysis for lung cancer risk

Table 3 lists the univariate and multivariate logistic regression analyses to determine the predictors of lung cancer. Female gender (univariate model: OR = 10.149, 95% confidence interval (CI) = 1.351–76.227; multivariate model: OR = 11.199, 95% CI = 1.444–86.862), nodule number (univariate model: OR = 1.353, 95% CI = 1.114–1.642; multivariate model: OR = 1.309, 95% CI = 1.066–1.607), and family history of lung cancer (univariate model: OR = 3.08,95% CI = 1.108–8.557; multivariate model: OR = 2.831, 95% CI = 1.000136–8.015) were significant associated with lung cancer both on univariate and multivariate analysis.

**Table 3:** Univariate and multivariate logistic regression analyses for predictors of lung cancer in 784 subjects after propensity score matching

	Univariate analysis			Multivariate analysis		
Characterics	Odds ratio	95% CI	*P* value	Odds ratio	95% CI	*P* value
Age, years	1.015	0.953–1.082	0.641	0.994	0.923–1.070	0.871
Sex (female gender)	10.149	1.351–76.227	0.024	11.199	1.444–86.862	0.021
BMI, kg/m^2^	1.015	0.895–1.151	0.815	1.079	0.953–1.221	0.23
Nodule number	1.353	1.114–1.642	0.02	1.309	1.066–1.607	0.01
Family history of lung cancer	3.08	1.108–8.557	0.031	2.831	1.000136–8.015	0.05
Family history of other cancer	1.241	0.501–3.073	0.641	1.078	0.425–2.732	0.875

Abbreviations: *BMI* body mass index, *CI* confidence interval

## Discussion

**Fig. 3:**
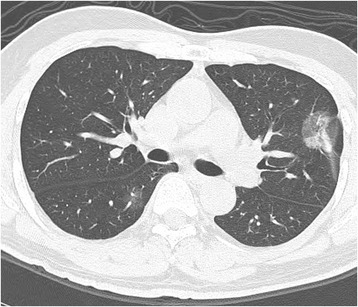
An example of multifocal GG/L lung cancer, a kind of lung adenocarcinoma subtype which often occurred in Asian women or non-smoker recently according to the IASLC Lung Cancer Staging Project in 2016. A 61-year-old woman had a 2.8 cm part-solid nodule in LUL, and another one pure GGN nodule 1.4 cm in RLL. The patient underwent sequentially video-thoracoscopic wedge resection of RLL and LUL. Further pathologic report demonstrated invasive adenocarcinoma in LUL, and adenocarcinoma in situ in RLL. Synchronous multiple primary lung cancer was diagnosed according to the diagnostic criteria proposed by Martini and Melamed. Abbreviations: RLL = right lower lobe; GGN = groundglass nodule; LUL = left upper lobe

In this retrospective analysis applying propensity score matching in order to minimize confounding effects and selection bias to estimate the true causal effect, we demonstrated three major findings. The first one is that to utilize the propensity score matching to adjust for selection bias could address the balanced baseline characteristics between exposure and control subjects and improve the internal validity of the study. The second finding is family history of lung cancer and female gender were significantly associated with lung cancer based on univariate or multivariate logistic regression. Previous studies have addressed the issue that family history of lung cancer significantly association with non-smoking related lung cancer, mainly in middle-age women of Asian population. However, these results were based on data available from previous case-control or retrospective cohort studies which more susceptible to the effects of selection bias [7, 8]. The present study demonstrated for the first time that identification two important associated risk factors with lung cancer in an Asian cohort with less smoker using a propensity score matching method to construct quasi-experimental design intended to stimulate randomized controlled trial (RCT) design and minimize the selection bias [17]. Familial risk of lung cancer is attributable to share more complex genetic and environmental factors [18–20]. Our study demonstrated that familial history of lung cancer significantly associated with non-smoking related lung cancer, especially in women. In addition, another study demonstrated that women with a history of lung infection (bronchitis or pneumonia) positively influenced lung cancer development [21]. The third finding, increasing numbers of nodules were significantly associated with lung cancer in an Asian population, mainly non-smoker. The reported incidence of synchronous MPLCs in patients with lung cancer in our study is high up to 35% (one example case shown in Fig. 3). The incidence of synchronous MPLC has been reported to range from 0.7% to 30% of patients with lung cancer in the previous literature reviews [8, 22–24]. This study result support that high prevalence of Multifocal ground glass/lepidic (GG/L) lung cancer, a kind of lung adenocarcinoma subtype which often occurred in Asian women or non-smoker recently proposed by the International Association for the Study of Lung Cancer (IASLC) Lung Cancer Staging Project in 2016 [9]. In addition, the overall lung cancer prevalence rate was 1.40% (27/1975) in this study cohort. Our results are congruent with other published data from Asian population in the group of non-smokers or lesser smokers (lung cancer prevalence rate 1 ~ 2% at the baseline LDCT screening) [25, 26]. Our study population consists mainly of non-smokers, which is very different from the NLST and other LDCT lung cancer screening studies conducted outside of Asia [3, 27]. Recent studies have investigated more detail about the diagnosis, management and prognosis of Multifocal GG/L lung cancer [28–30]. This issue should be more emphasized in Asian lung cancer screening program due to high prevalence of synchronous MPLC reported according to previous and the current studies [8, 22].

There are several limitations to our study. First, propensity score matching can only control for observed covariates such as age, BMI or sex in the study. However, any unobserved covariates (cooking, second-hand smoking and air pollution) cannot be adjusted to balancing baseline characteristics between exposure and unexposed with reducing selection bias [31]. Second, propensity score matching methods resulted in throwing out over half of the subjects in the unexposed group, reducing the overall sample size and negatively affecting statistical power. To maximize our statistical power to detect this effect, it is mandatory to perform a much larger cohort in an Asian population. Third, a large number of subjects are eliminated after propensity scoring matching because of limited numbers within the exposure group despite the algorithm of full matching. Thus further large cohort studies are needed to establish generalizability of these study results because of the loss of study subjects numbers threatening external validity.

## Conclusion

In conclusion, in this retrospective analysis applying propensity score matching in order to minimize confounding effects and identify two important risk factors of female gender and family history of lung cancer for non-smoker lung cancer prediction. In the future, risk-based prediction model based on the family history of lung cancer and female gender can potentially improve efficiency of lung cancer screening programs in Taiwan.

## Abbreviations

ACR: American College of Radiology
BMI: Body mass index
CI: Confidence interval
CMS: Centers for Medicare & Medicaid Services
CT: Computed tomography
CTDIvol: Volume CT dose index
GG/L: Ground glass/lepidic
IASLC: International Association for the Study of Lung Cancer
LDCT: Low-dose computed tomography
Lung-RADS: Lung Imaging Reporting and Data System
MPLC: Multiple primary lung cancers
NLST: National Lung Screening Trial
OR: Odds ratio
RCT: Randomized controlled trial
SD: Standard deviation
